# Influence of Retirement on Adherence to Statins in the Insurance Medicine All-Sweden Total Population Data Base

**DOI:** 10.1371/journal.pone.0130901

**Published:** 2015-06-23

**Authors:** Heli Halava, Hugo Westerlund, Maarit Jaana Korhonen, Jaana Pentti, Mika Kivimäki, Linnea Kjeldgård, Kristina Alexanderson, Jussi Vahtera

**Affiliations:** 1 Department of Public Health, University of Turku, Turku, Finland; 2 Stress Research Institute, Stockholm University, Stockholm, Sweden; 3 Department of Clinical Neuroscience, Karolinska Institutet, Stockholm, Sweden; 4 Department of Pharmacology, Drug Development and Therapeutics, University of Turku, Turku, Finland; 5 Finnish Institute of Occupational Health, Turku, Finland; 6 Department of Epidemiology and Public Health, University College of London, London, United Kingdom; 7 Turku University Hospital, Turku, Finland; University of Bologna, ITALY

## Abstract

**Background:**

Retirement has been suggested to reduce medication adherence, but no evidence is available for statins. We investigated changes in adherence to statins among Swedish adults after retirement.

**Methods:**

A prospective cohort study was carried out on all individuals living in Sweden on 31 December 2004, alive in 2010, having purchased statins in the second half of 2005, and retired in 2008 (n=11 718). We used prescription dispensing data in 2006–2010 to determine nonadherence (defined as <80% of days covered by filled prescriptions) before and after old-age or disability retirement. Using multiple repeat measurements of filled statin prescriptions, we calculated the annual prevalence rates of nonadherence for those who continued therapy. Discontinuation was defined as no statin dispensations during a calendar year.

**Results:**

After adjustment for age at retirement, the prevalence ratio (PR) of nonadherence after retirement in comparison with those before retirement was 1.23 [95% confidence interval (CI) 1.17–1.29] for the men and 1.19 (95% CI 1.13–1.26) for the women. A post-retirement increase in nonadherence was consistently observed across the strata of age at retirement, marital status, education, income, type of retirement, and participants with and without cardiovascular disease, the largest increases being observed for statin use in secondary prevention (men: PR 1.38, 95% CI 1.26–1.54; women: PR 1.43, 1.18–1.72). For primary prevention, the corresponding prevalence ratios were 1.18 (95% CI 1.13‒1.25) and 1.18 (95% CI 1.11–1.24), respectively.

**Interpretation:**

Retirement appears to be associated with increased nonadherence to statin therapy among Swedish men and women.

## Introduction

Cardiovascular disease is the leading cause of death worldwide. In Europe, there are almost 4.1 million deaths/year; of these, ~1.8 million people die of coronary heart disease and ~1.1 million of stroke.[1] The major cardiovascular risk factors include high blood pressure, smoking, unhealthy diet, and high total cholesterol.[2] Randomized controlled trials have provided convincing evidence on the benefits of statin therapy in preventing cardiovascular events.[3] However, over 40% of the patients prescribed statins are nonadherent, consuming less than 80% of the prescribed medication. This nonadherence translates to 9 extra cases of major cardiovascular events/100 000 individuals annually.[4] Therefore, identifying factors that affect adherence form a major public health challenge.

Much of the research on medication adherence has focused on its associations with patients’ demographic and clinical characteristics, physicians’ and pharmacists' performance, and the functioning of health systems.[5] For instance, a patient’s age, gender, and socioeconomic status may be related to adherence.[6,7] Moreover, symptomatic patients and those with severe diseases [8] may have better adherence than those on preventive medication for asymptomatic conditions, such as hypertension or dyslipidemia.[9]

Recent studies suggest that such life transitions as retirement, for example, may also affect adherence to medication.[10] In a study of Finnish public-sector employees, for instance, a decline in medication adherence was observed after retirement among men and women with hypertension and among men with type-2 diabetes.[11] One potential explanation is a perception of fewer symptoms of ill health after retirement [12], the result being less motivation to take medication for asymptomatic conditions. Although hypercholesterolemia is a common asymptomatic condition, we are aware of no studies that have examined changes in adherence to statins after retirement.

The aim of this study was to determine whether retirement is associated with changes in nonadherence to statins. As some people retire early due to illness and others retire on old-age pensions [13], we also took into account the type of retirement.

## Materials and Methods

A prospective cohort study was conducted. We employed multiple repeat measurements of filled statin prescriptions, both before and after retirement, to determine whether retirement was associated with a change in nonadherence to statins among patients in the prevention of cardiovascular disease in the total population. (Insurance Medicine All-Sweden database, [14])

### Study population and design

**Fig 1:**
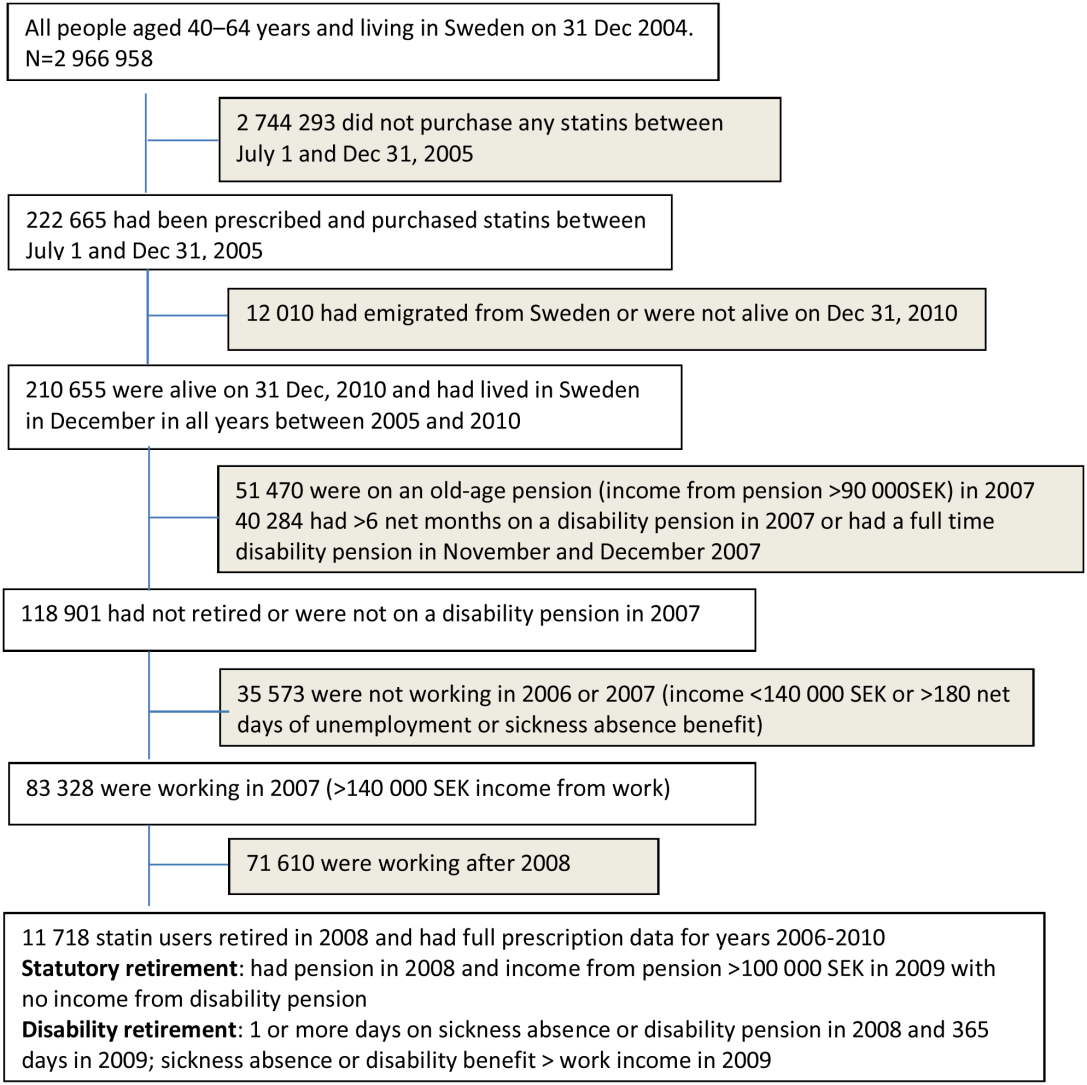
Flow chart of sample selection.

From the Insurance Medicine All-Sweden database, we first selected all individuals aged 40–64 years (n = 2 966 958) and living in Sweden on 31 December 2004. Those who did not fill any prescriptions for statins between 1 July and 31 December 2005 were excluded (n = 2 744 293). Those who filled one or more statin prescriptions during the last 6 months of 2005, but emigrated from Sweden or had died by 31 December 2010, or had retired or had no income from work in 2007 were excluded (n = 12 010). Of the remaining individuals, we selected all those granted a disability pension or retired in 2008 (Fig 1). Thus the final cohort comprised 11 718 statin users who had lived in Sweden between 2005 and 2010, were at work until 2007, retired in 2008, and for whom data on filled statin prescriptions were available for the whole period of 2006–2010. The regional Ethical Review Board of Stockholm, Sweden, approved the study.

### Data sources

Register data from three authorities were linked. Statistics Sweden provided gender, age, educational level, marital status (married or registered partnership: yes or no), annual income, retirement, and emigration. The National Social Insurance Agency provided the beginning date of disability pensions. The National Board of Health and Welfare provided data from its patient register (dates and diagnoses according to the 10^th^ version of the International Classification of Diseases [ICD-10,[15]] for hospitalizations and hospital-based out-patient care), the Cause of Death Register, and the Swedish Prescribed Drug Registry. Data from these different registers were linked by means of the personal identification numbers attributed to all people living in Sweden.

### Outcome

The primary outcome variable was nonadherence to statins among patients not discontinuing their therapy in the 5-year observation period, centered on disability pension/retirement in 2008. The secondary outcome was discontinuation of statin therapy.

Information on statin use (July 2005-December 2010) came from the Prescribed Drug Registry. For each dispensed drug, information on the Anatomical Therapeutic Chemical (ATC) code [16], the date of dispensation, and the quantity dispensed were available. In Sweden, statins (ATC code C10AA) are available by prescription only.

For each calendar year of follow-up, starting on 1 January 2006, adherence was defined as the proportion of days covered (PDC) by the dispensed tablets multiplied by 100, on the assumption of a dosage of 1 tablet per day.[17] Switching between various statins was considered to be a continuation of therapy. Consistent with previous research, we defined nonadherence (PDC <80%) using the conventional cut-point for dichotomy. Adherent use refers to PDC ≥80%. Although somewhat arbitrary, this is the most widely used cut-point for optimum adherence.[4]

Discontinuation of statin therapy was defined as no statin dispensations during a calendar year.[18]

### Covariates

The educational level was classified as high (≥12 years; university education), intermediate (10–12 years; upper secondary school), or basic (≤9 years; compulsory school or unknown), marital status (married or registered partnership in 2007) as yes or no, and income in 2007 as low (<250 000 Swedish Kronor SEK [1 SEK≈0.11 EUR, April 2015]/year = below existence minimum) or high (≥250 000 SEK/year). Type of retirement was classified as statutory or disability pension. In Sweden, the ordinary retirement age is 65 years, but a pension can be taken earlier. All adults below the age of 65 years who have a permanently reduced work capacity due to disease or injury can be granted a disability pension.

The type of prevention was determined according to inpatient and outpatient hospital visits due to coronary heart or cerebrovascular diseases (ICD-10 codes I20-I25 and I60-I69) in any year before retirement. Statin treatment was considered to be primary prevention if an individual had had no such visits and as secondary prevention otherwise.

### Statistical analyses

The analyses were based on a 5-year observation period for medication adherence, including 2 years before and 2 years after the year of retirement. Data were stratified by gender because of gender differences in cardiovascular disease rates[1], in adherence to cardiovascular medication[6,19], in early and old-age retirement[20–22], and in the effects of illnesses and life transitions on adherence to some cardio-preventive medications.[11,23] Analyses were performed separately for those with at least one statin dispensation during a calendar year (continuers) and those with no dispensations (discontinuers). We calculated the annual prevalence (95% confidence intervals [CI]) of nonadherence and discontinuation using a repeated-measures log-binomial regression with the generalized estimating equations (GEE) method to account for the intra-individual correlation between measurements.[24] In these models, we used contrasts to estimate the change in the mean prevalence of nonadherence among those not discontinuing statin therapy in the 2 years after retirement in comparison with the 2 years before retirement, adjusted for age at retirement. In relation to the discontinuation of statin therapy, we used log-binomial regression with the generalized estimating equations (GEE) and contrasted the prevalence in the last year of follow-up to the prevalence in the first year, as there was no apparent change in the trend around retirement.

To examine whether the associations varied across subgroups (i.e., age, educational level, marital status, income, type of retirement [statutory or disability pension], and type of prevention [primary or secondary]), we calculated the prevalence ratio (PR) of nonadherence after retirement in comparison with that before retirement from models including the subgroup, time, calendar year, age at retirement, and the interaction term “subgroup*time”.

To evaluate potential confounding due to a change in the reimbursement regulations in 2009[25], we replicated the main analyses using nonadherence to simvastatin (ATC code C10AA01) as the outcome and compared the trends in nonadherence between simvastatin and all statins. The new reimbursement scheme for lipid-modifying therapies was assumed not to have affected simvastatin use appreciably since most of the simvastatin users received during the study [26] generic simvastatin, fully reimbursed also under the new reimbursement scheme. [25]

All of the statistical analyses were conducted with SAS 9.2 statistical software (SAS Institute, Inc., Cary, North Carolina).

### Ethical approval

Patient information was anonymized and de-identified prior to analysis. The regional Ethical Review Board of Stockholm, Sweden, approved the study.

## Results

The 11 718 retiring statin users were predominantly men (59.2%). Table 1 shows the baseline characteristics of the cohort. The mean age at retirement was 62.7 years (standard deviation [SD] 3.6 years) for the men and 62.3 years (SD 4.0 years) for the women. Of the men, one-third had a low educational level and a low income; of the women, one-fifth had a low educational level, but two-thirds had a low income. About two-thirds of both genders were married. A quarter of the men and one-third of the women had retired early due to ill health. Most (72.5% of the men and 88.5% of the women) used statins for primary prevention. Altogether 16.5% of the men and 20.6% of the women were nonadherent in 2006. The corresponding proportions were 17.8% and 21.7% for primary prevention and 12.9% and 13.3% for secondary prevention, respectively. S1 Table provides the figures for discontinuation in the first and last year of follow-up.

**Table 1:** Descriptive statistics of the study population of 11 718 men and women.

	Men (59.2% of all)		Women (40.8% of all)	
Characteristic	n (%)	Nonadherence^a^ prevalence (%)in 2006	n (%)	Nonadherence^a^ prevalence (%)in 2006
All	6938 (100.0)	16.5	4780 (100.0)	20.6
**Retirement age (years)**				
44–63	3196 (46.1)	17.2	2380 (49.8)	21.5
64–68	3742 (53.9)	15.9	2400 (50.2)	20.1
**Educational level**				
Compulsory school	2149 (31.0)	16.0	939 (19.6)	19.2
Upper secondary school	3054 (44.0)	16.4	2089 (43.7)	19.7
University education	1735 (25.0)	17.1	1752 (36.7)	22.7
**Married**				
Yes	4881 (70.4)	15.7	3004 (62.9)	19.7
No	2057 (29.7)	18.2	1776 (37.2)	22.5
**Income (SEK/year)**				
<250 000	2390 (34.5)	16.6	2946 (61.6)	19.3
≥250 000	4548 (65.6)	16.4	1834 (38.4)	22.9
**Type of retirement**				
Statutory	5144 (74.1)	16.7	3116 (65.2)	20.4
Disability	1794 (25.9)	15.9	1664 (34.8)	21.3
**Type of prevention**				
Primary	5033 (72.5)	17.8	4232 (88.5)	21.7
Secondary^b^	1905 (27.5)	12.9	548 (11.5)	13.3

^a^Nonadherence refers to proportion of days covered by treatment <80% for patients not discontinuing their therapy. The year 2006 was two years before the year of retirement/ disability pension.

^b^Secondary prevention: previous in- or outpatient hospital visits due to coronary heart disease or cerebrovascular diseases in any year before retirement.

### Retirement and nonadherence among the men

**Fig 2:**
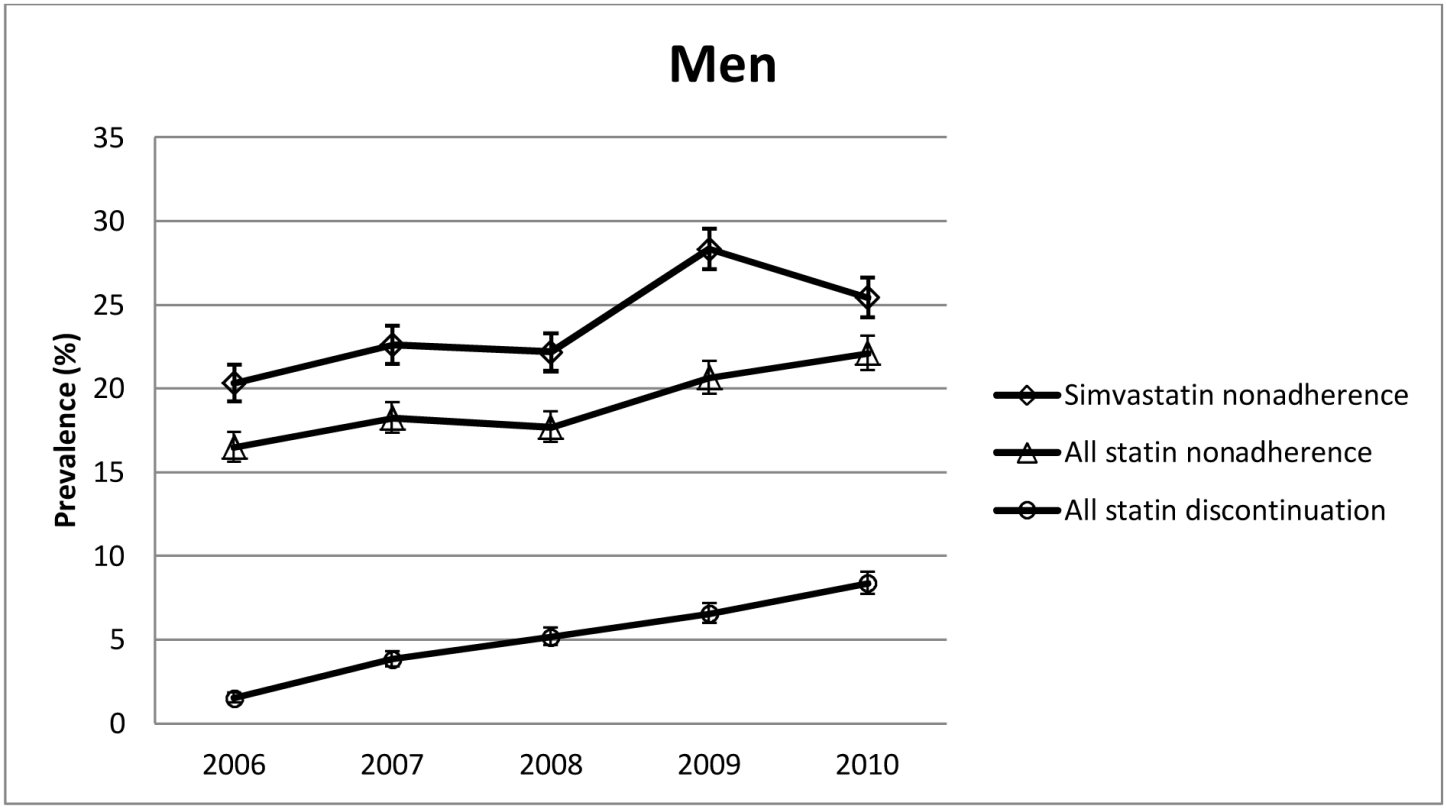
Nonadherence and discontinuation prevalences in men.

When adjusted for age at retirement, the nonadherence prevalence of the men who did not discontinue their statin therapy remained at about the same level until the year of retirement. After 2008, there was a step-like increase, the prevalence of nonadherence increasing from 17.7% in 2008 to 22.1% in 2010 (Fig 2). When compared with the level 2 years before the year of retirement, the prevalence of nonadherence (adjusted for age at retirement) was 1.23 (95% CI 1.17–1.29) times higher in the 2 years after retirement. Higher post-retirement nonadherence was observed in all of the subgroups (Table 2).

**Table 2:** Change in the prevalence of nonadherence to statins after retirement in the patient subgroups not discontinuing their therapy.

	Nonadherence^a^ after vs. before retirementPrevalence ratio^b^(95% Confidence interval)	
Characteristic	Men	Women
All	1.23 (1.17–1.29)	1.19 (1.13–1.26)
**Retirement age (years)**		
44–63	1.22 (1.14–1.31)	1.23 (1.14–1.32)
64–68	1.24 (1.16–1.32)	1.16 (1.08–1.25)
**Educational level**		
Compulsory school	1.20 (1.11–1.31)	1.14 (1.01–1.29)
Upper secondary school	1.21 (1.13–1.30)	1.22 (1.12–1.31)
University education	1.30 (1.18–1.42)	1.19 (1.00–1.30)
**Married**		
Yes	1.23 (1.13–1.34)	1.12 (1.03–1.22)
No	1.23 (1.17–1.31)	1.25 (1.17–1.33)
**Income (SEK/year)**		
<250 000	1.20 (1.11–1.30)	1.23 (1.15–1.31)
≥250 000	1.25 (1.17–1.32)	1.15 (1.06–1.25)
**Type of retirement**		
Statutory	1.24 (1.17–1.31)	1.16 (1.08–1.24)
Disability	1.20 (1.10–1.31)	1.26 (1.16–1.37)
**Type of prevention**		
Primary	1.18 (1.13–1.25)	1.18 (1.11–1.24)
Secondary ^c^	1.38 (1.26–1.54)	1.43 (1.18–1.72)

Prevalence ratios were derived from repeated measures log-binomial regression analyses adjusted for age at retirement.

^a^Nonadherence refers to proportion of days covered by treatment <80%.

^b^Prevalence ratio for nonadherence in the 2 years after retirement compared with the 2 years before retirement.

^c^ Secondary prevention: previous in- or outpatient hospital visits due to coronary heart disease or cerebrovascular diseases in any year before retirement.

The only significant (P for interaction 0.048) difference in the relative increase in post-retirement nonadherence between the subgroups was observed for type of prevention, the PR being 1.38 (95% CI 1.26–1.54) for secondary prevention and 1.18 (95% CI 1.13–1.25) for primary prevention.

### Retirement and nonadherence among the women

**Fig 3:**
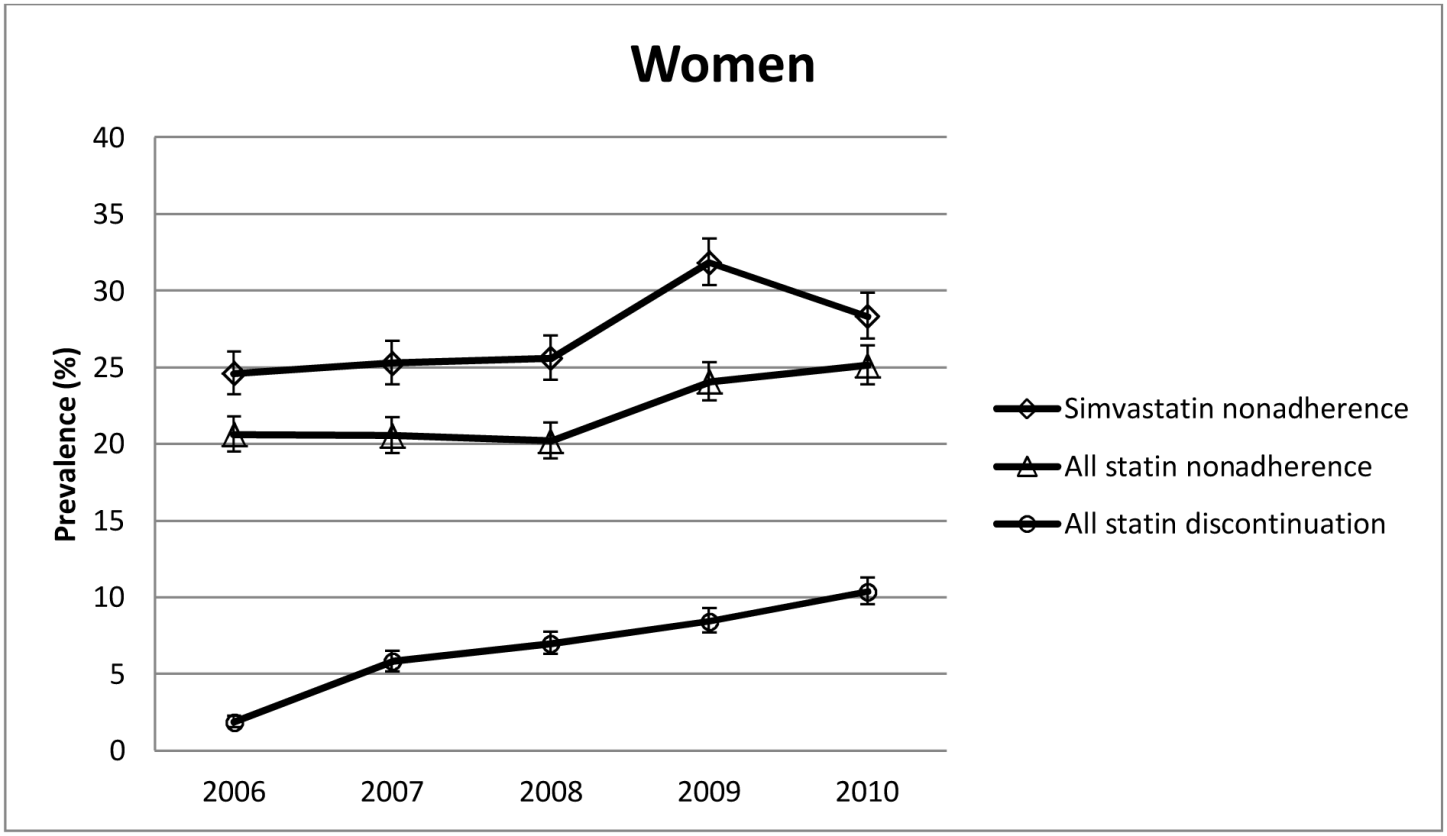
Nonadherence and discontinuation prevalences in women.

Among the women, there was a similar, step-like increase in the nonadherence prevalence from 20.2% in 2008 to 25.1% in 2010 (Fig 3) after retirement. The PR for nonadherence after versus before retirement was 1.19 (95% CI 1.13–1.26) when adjusted for age at retirement. This significantly higher post-retirement nonadherence was observed for all the subgroups (all interactions between subgroup and time: P-values ≥0.19). (Table 2) Also among the women, the highest increase in the prevalence of nonadherence was observed among those receiving statins for secondary prevention (PR 1.43, 95% CI 1.18–1.72); for primary prevention, the corresponding PR was 1.18 (1.11–1.24).

### Retirement and discontinuation

In 2006, 1.5% of the men and 1.9% of the women discontinued statin therapy. In 2010, these discontinuation prevalences were 8.4% and 10.4%, respectively. As shown in Figs 2 and 3, this increase in discontinuation was linear for both genders with no apparent change in the trend around retirement.

Table 3 presents the prevalence of discontinuation in the last year of follow-up in comparison with that of the first year by gender and subgroup. The proportion of discontinuers among the men was 4.5–6.5 times higher, and among the women it was 4.3–10.3 times higher, in 2010 than in 2006, depending on the subgroup (S1 Table).

**Table 3:** Change in the prevalence of discontinuation^a^ of statin therapy in the patient subgroups during the 5-year exposure period.

	Prevalence ratio^b^(95% Confidence interval)	
Characteristic	Men	Women
All	5.53 (4.58–6.69)	5.50 (4.48–6.76)
**Retirement age (years)**		
44–63	5.20 (4.00–6.78)	4.91 (3.78–6.38)
64–68	5.88 (4.48–7.72)	6.46 (4.64–8.99)
**Educational level**		
Compulsory school	4.53 (3.31–6.20)	10.33 (5.43–19.65)
Upper secondary school	6.50 (4.72–8.95)	4.36 (3.32–5.73)
University education	5.59 (3.91–7.98)	5.94 (4.18–8.44)
**Married**		
Yes	4.71 (3.50–6.32)	5.37 (3.92–7.35)
No	6.06 (4.74–7.76)	5.60 (4.28–7.34)
**Income (SEK/year)**		
<250 000	5.68 (4.11–7.85)	4.74 (3.70–6.07)
≥250 000	5.46 (4.32–6.90)	7.07 (4.93–10.15)
**Type of retirement**		
Statutory	5.66 (4.51–7.10)	6.77 (5.07–9.07)
Disability	5.23 (3.70–7.40)	4.26 (3.19–5.69)
**Type of prevention**		
Primary	5.46 (4.44–6.73)	5.49 (4.45–6.78)
Secondary ^c^	5.85 (3.70–9.23)	5.66 (2.43–13.20)

Repeated measures log-binomial regression analyses adjusted for age at retirement.

^a^ No purchases during a calendar year.

^b^The prevalence ratios of discontinuation are derived from contrasting the prevalence in the last year of follow-up to the prevalence in the first year

^c^ Secondary prevention: previous in- or outpatient hospital visits due to coronary heart disease or cerebrovascular diseases in any year before retirement.

### Sensitivity analysis: nonadherence to simvastatin

For a sensitivity analysis, we examined changes in nonadherence to simvastatin, a statin assumed to be the least affected by the change in the reimbursement regulations in 2009. The step-like post-retirement increase in the prevalence of nonadherence to simvastatin was similar to the increase in nonadherence to all statins among both the women and men (Figs 2 and 3) and in all of their subgroups (S2 Table).

## Discussion

In this population-based prospective cohort study, we found that the prevalence of nonadherence increased ~20% after retirement among both the men and the women. We observed no significant differences in this nonadherence pattern between the age groups, socioeconomic strata, or types of retirement. Our results were also robust when we restricted the analyses to the users of simvastatin, a statin which was not appreciably affected by the new reimbursement scheme for lipid-modifying therapies in Sweden in 2009. As our study is based on a total population, the patterns found suggest that the findings are generalizable to Sweden and not limited to a specific subgroup.

In men the only significant difference in the relative increase in post-retirement nonadherence between the subgroups was observed for type of prevention (test of interaction P = 0.048). The relative increase in nonadherence was twice as high in relation to secondary prevention (PR 1.38) as to primary prevention (PR 1.18). Among the women, these prevalent ratios were almost the same (1.43 and 1.18, correspondingly) but the confidence intervals overlapped (test for interaction P = 0.317).

### Comparison with other studies

Our finding of increased nonadherence to statins after retirement is in agreement with the scarce literature available. A recent study from Finland [11], which is a country with a similar single universal prescription reimbursement system and comprehensive prescription register as in Sweden, reported 1.3–2.4-fold post-retirement increases in the nonadherence prevalences of men and women with hypertension and of men with type 2 diabetes. As in our study, these changes in the adherence pattern were similar in all of the subgroups.

The reasons for an increase in post-retirement nonadherence are not known. Major life changes have been found to be associated with a lower adherence to medication[10] and a higher risk of depression,[27,28] although retirement is rated as being among the least severe of life events.[29] The change in daily routines after retirement may temporarily affect adherence; however, in this study, the level of adherence did not return to the level observed prior to retirement even during the second year of retirement. Previous research has consistently shown that patients with history of cardiovascular events, hypertension, or diabetes have better adherence to statin therapy than individuals free of these conditions.[30],[31],[32] Thus, cardiovascular comorbidities increasing with age could improve adherence. However, the fact that a step-like increase in nonadherence was found after retirement suggests that this nonadherence is unlikely to be explained by comorbidities. In our study the prevalence of nonadherence was lower in the secondary prevention group than in the primary prevention group, which is consistent with findings of previous studies.[6,9] Notably, the prevalence of nonadherence after retirement tended to increase relatively more in the secondary prevention group than in the primary prevention group regardless of the greater need for treatment in this group.

Also other indicators of health problems have been previously shown to associate with adherence to cardiovascular medications. For example, depression has been shown to decrease adherence to various preventive treatments, including statins and antihypertensive drugs [23,33]. However, it has been shown that retirement may improve mental health among old-age retirees [34,35] so this would be an unlikely explanation for post-retirement nonadherence.

Previous studies have found that geography might also affect adherence behaviour. For example in heart failure patients clinically important differences in medication use between rural and urban patients exist but mainly in selective prescribing of different heart failure medications. Only few differences in adherence were noted between rural and urban patients.[36] A Danish population-based study [37] found that patients who discontinued their statin therapy were more likely to live in small municipalities in rural areas and more likely to be divorced, whereas another study [38] found no consistent evidence that bereavement affects negatively the probability that a patient has a higher than 80% adherence with medication rate. Thus, changes in living conditions following retirement would probably have only a small effect on adherence.

The observed rates of discontinuation in this study were not directly comparable to those previously reported because of differences in definitions of discontinuation and because our study population consisted of prevalent statin users, not initiators. According to previous studies the discontinuation rate for statin medication is the steepest within the first year after initiation.[39] Within 2 years, the discontinuation rate has been shown to decrease to an annual level of 1.5%.[40] Our finding of a linear increase in discontinuation among prevalent users is in line with the results of previous research.

### Strengths and limitations

Our large, prospective, total population-based study included all people fulfilling the inclusion criteria. Furthermore, the study covered several years, had no losses to follow up, and used only data from several nationwide registers of good quality. Sweden is a particularly favorable setting for pharmaco-epidemiological studies due to its legislation, and the completeness and accuracy of its pharmacy records are high as all information about drugs dispensed and purchased by patients is entered into a national database. Due to the availability of statins in Sweden by prescription only, the Prescription Drug Registry provided comprehensive data on statin purchases.

One limitation of this study was the inability of the refill measure to capture nonadherence when the dispensed medications were not used.

Another limitation was that, due to reliance on register data, we did not have data on biological and lifestyle factors that may affect adherence to cardio-preventive medications. [41]

It is possible that unmeasured, time-dependent confounders occurring at the time our participants retired could explain the increase in nonadherence. One potential confounder is the new reimbursement scheme for lipid-modifying therapies that was implemented in Sweden in June of 2009.[25] In the new scheme, the reimbursement for statin products on the market was either continued (generic pravastatin, generic simvastatin, and from branded simvastatin 80 mg in packs of 49 tablets), restricted (atorvastatin in strengths of >10 mg and rosuvastatin in strengths >5 mg) or discontinued (fluvastatin, branded pravastatin, branded simvastatin, atorvastatin 10 mg and rosuvastatin 5 mg). After the implementation of the new reimbursement scheme, lipid-modifying treatment patterns changed dramatically: among patients initially treated with lower doses of rosuvastatin or atorvastatin the discontinuation rates doubled after the implementation and switching to higher doses increased. However, among the users of statins with continued full reimbursement (generic simvastatin and pravastatin), no statistically significant differences in the level or trend of treatment was observed after the implementation.

In our study, a similar, post-retirement increase in nonadherence was observed among those with continued full reimbursement (i.e. simvastatin users) than among all statin users. The coincident implementation of the new reimbursement scheme after the year of retirement thus is not likely to explain our findings.

## Conclusion

Retirement is a life transition, which can increase nonadherence to statins among men and women in primary and secondary prevention. Our finding of an increased nonadherence to statins after retirement is important because the proportion of people aged 65 years or older is growing rapidly. It is important for health professionals to recognize this post-retirement risk of nonadherence because nonadherence to statins is associated with an increased risk of adverse cardiovascular outcomes and higher healthcare costs. Possible reasons for this increase in nonadherence and interventions to improve adherence deserve further research.

## Supporting Information

S1 Fig Prevalence of nonadherence to statin medication in the first year (2006) of the follow-up and the increase in nonadherence prevalence from 2006 to 2010 by patient subgroup in men and in women. (DOCX)

S1 Table Prevalences (%) of discontinuation of statin therapy in the first (2006) and the last (2010) year of follow-up in the patient subgroups. (DOCX)

S2 Table Change in the prevalence of nonadherence to simvastatin after retirement in patient subgroups not discontinuing their therapy. (DOCX)
