# Supplementary material for: Influence of Retirement on Adherence to Statins in the Insurance Medicine All-Sweden Total Population Data Base
Source: PLoS One. 2015 Jun 23;10(6):e0130901. doi: 10.1371/journal.pone.0130901 (PMC4477901; doi:10.1371/journal.pone.0130901)
Supplement: S1 Fig — (DOCX) [file pone.0130901.s001.docx]

**S1 Fig.** Prevalence of nonadherence to statin medication in the first year (2006) of the follow-up and the increase in nonadherence prevalence from 2006 to 2010 by patient subgroup in men and in women.
