# Supplementary material for: Influence of Retirement on Adherence to Statins in the Insurance Medicine All-Sweden Total Population Data Base
Source: PLoS One. 2015 Jun 23;10(6):e0130901. doi: 10.1371/journal.pone.0130901 (PMC4477901; doi:10.1371/journal.pone.0130901)
Supplement: S1 Table — (DOCX) [file pone.0130901.s002.docx]

**S1 Table. Prevalences (%) of discontinuationᵃ of statin therapy in the first (2006) and the last (2010) year of follow-up in the patient subgroups.**

|  | **Men** | | **Women** | |
| --- | --- | --- | --- | --- |
| **Characteristic** | **First year %**  **(95% CI)** | **Last year %**  **(95% CI)** | **First year %**  **(95% CI)** | **Last year %**  **(95% CI)** |
| **All** | 1.5 (1.3‒1.8) | 8.4 (7.7‒9.1) | 1.9 (1.5‒2.3) | 10.4 (9.6‒11.3) |
| **Retirement age (years)** |  |  |  |  |
| 44‒63 | 1.7 (1.3‒2.2) | 8.8 (7.9‒9.8) | 2.4 (1.8‒3.0) | 11.6 (10.3‒12.9) |
| 64‒68 | 1.4 (1.0‒1.8) | 8.0 (7.2‒8.9) | 1.5 (1.0‒2.0) | 9.4 (8.3‒10.7) |
| **Educational level** |  |  |  |  |
| Compulsory school | 1.8 (1.3‒2.4) | 8.0 (7.0‒9.3) | 1.0 (0.5‒1.9) | 10.0 (8.2‒12.1) |
| Upper secondary school | 1.2 (0.9‒1.7) | 8.0 (7.1‒9.0) | 2.4 (1.8‒3.1) | 10.4 (9.1‒11.7) |
| University education | 1.7 (1.2‒2.4) | 9.3 (8.0‒10.7) | 1.8 (1.3‒2.6) | 10.8 (9.4‒12.4) |
| **Married** |  |  |  |  |
| Yes | 1.3 (1.0‒1.7) | 8.0 (7.2‒8.8) | 1.8 (1.3‒2.3) | 9.9 (8.9‒11.0) |
| No | 2.0 (1.4‒2.7) | 9.2 (8.1‒10.6) | 2.1 (1.6‒2.9) | 11.4 (10.0‒13.0) |
| **Income (SEK/year)** |  |  |  |  |
| <250 000 | 1.4 (1.0‒2.0) | 8.1 (7.0‒9.3) | 2.0 (1.6‒2.6) | 9.7 (8.6‒10.8) |
| ≥250 000 | 1.5 (1.2‒2.0) | 8.5 (7.7‒9.3) | 1.7 (1.2‒2.4) | 11.7 (10.3‒13.3) |
| **Type of retirement** |  |  |  |  |
| Statutory | 1.5 (1.2‒1.9) | 8.5 (7.7‒9.4) | 1.5 (1.1‒1.9) | 9.8 (8.7‒11.1) |
| Disability | 1.5 (1.0‒2.1) | 7.8 (6.5‒9.4) | 2.7 (2.0‒3.7) | 11.7 (9.9‒13.8) |
| **Type of prevention** |  |  |  |  |
| Primary | 1.7 (1.4‒2.1) | 9.2 (8.4‒10.0) | 2.0 (1.6‒2.5) | 11.0 (10.1‒12.0) |
| Secondaryᵇ | 1.0 (0.7‒1.6) | 6.0 (5.1‒7.2) | 1.0 (0.5‒2.3) | 5.9 (4.3‒8.2) |

ᵃ No purchases during a calendar year.

ᵇSecondary prevention: previous in- or outpatient hospital visits due to coronary heart disease or cerebrovascular diseases in any year before retirement.
