# Supplementary material for: Influence of Retirement on Adherence to Statins in the Insurance Medicine All-Sweden Total Population Data Base
Source: PLoS One. 2015 Jun 23;10(6):e0130901. doi: 10.1371/journal.pone.0130901 (PMC4477901; doi:10.1371/journal.pone.0130901)
Supplement: S2 Table — (DOCX) [file pone.0130901.s003.docx]

**S2 Table. Change in the prevalence of nonadherence to simvastatin after retirement in patient subgroups not discontinuing their therapy.**

|  | **Nonadherenceᵃ after vs. before retirement**  **Prevalence ratioᵇ**  **(95% Confidence interval)** | |
| --- | --- | --- |
| **Characteristic** | **Men** | **Women** |
| All | 1.25 (1.19‒1.31) | 1.21 (1.14‒1.27) |
| **Retirement age (years)** |  |  |
| 44‒63 | 1.23 (1.15‒1.32) | 1.24 (1.15‒1.33) |
| 64‒68 | 1.27 (1.19‒1.36) | 1.17 (1.08‒1.26) |
| **Educational level** |  |  |
| Compulsory school | 1.26 (1.16‒1.38) | 1.23 (1.09‒1.40) |
| Upper secondary school | 1.21 (1.12‒1.30) | 1.18 (1.09‒1.28) |
| University education | 1.32 (1.20‒1.46) | 1.22 (1.12‒1.33) |
| **Married** |  |  |
| Yes | 1.23 (1.13‒1.34) | 1.12 (1.03‒1.22) |
| No | 1.27 (1.20‒1.34) | 1.26 (1.18‒1.35) |
| **Income (SEK/year)** |  |  |
| <250 000 | 1.24 (1.14‒1.34) | 1.24 (1.16‒1.33) |
| >250 000 | 1.26 (1.19‒1.34) | 1.16 (1.07‒1.25) |
| **Type of retirement** |  |  |
| Statutory | 1.27 (1.20‒1.34) | 1.18 (1.11‒1.26) |
| Disability | 1.22 (1.11‒1.33) | 1.25 (1.15‒1.36) |
| **Type of prevention** |  |  |
| Primary | 1.21 (1.14‒1.27) | 1.18 (1.12‒1.25) |
| Secondary ͨ | 1.38 (1.25‒1.53) | 1.40 (1.17‒1.67) |

Prevalence ratios derived from repeated measures log-binomial regression analyses adjusted for age at retirement.

ᵃNonadherence refers to proportion of days covered by treatment <80%.

ᵇPrevalence ratio for nonadherence in the 2 years after retirement compared with the 2 years before retirement.

ͨ Secondary prevention: previous in- or outpatient hospital visits due to coronary heart disease or cerebrovascular diseases in any year before retirement.
